# A Longitudinal Approach to the Relationships Among Sleep, Behavioral Adjustment, and Maternal Depression in Preschoolers

**DOI:** 10.3389/fpsyg.2022.819657

**Published:** 2022-04-13

**Authors:** Kijoo Cha

**Affiliations:** Department of Early Childhood Education, Gachon University, Seongnam, South Korea

**Keywords:** sleep duration, sleep problems, child adjustment, maternal depression, trajectory, preschoolers

## Abstract

This study aimed to investigate the longitudinal associations between children’s sleep duration (SD) and problems (SPs), behavioral adjustment [externalizing behaviors (EB) and internalizing behaviors (IB)], and maternal depressive symptoms (MDS) in preschoolers over a period of 3 years (4–6 years of age). For this purpose, latent growth modeling (LGM) was conducted using 2012(W_5_) to 2014(W_7_) data from the National Panel Study on Korean Children (PSKC), while controlling for family contextual factors (i.e., responsive parenting, developmental stimulations, and marital conflict) and child temperament (children’s negative emotionality). First, children who slept longer at four were concurrently associated with lower levels of EB, while more SPs were associated with higher levels of EB and IB, concurrently. Second, greater decreases in SPs were associated with greater decline in EB and IB. Higher levels of MDS at four were associated with higher levels of child EB, IB, and SPs, concurrently. However, no longitudinal associations were found between the rates of change in MDS and children’s sleep and adjustment (EB and IB). Finally, the magnitude of the associations among the variables was greater overall in the SPs models than in the SD models. These findings suggest that addressing sleep problems, rather than sleep duration, seem to be more important in predicting and preventing young children’s adjustment problems and also that more attention should be paid to MDS during preschool years as much as during the postpartum period for better child adjustment outcomes.

## Introduction

Like many other aspects of human development, sleep undergoes dramatic changes during the early years of life, both quantitatively and qualitatively. Compared to adults, young children spend substantially more time sleeping (Thorleifsdottir et al., 2002; Spruyt et al., 2005; Galland et al., 2012). In general, newborns sleep up to 18 h a day, which gradually decreases to approximately 10–12 h (42%–46% decrease) by the time elementary school begins (Thorleifsdottir et al., 2002; Spruyt et al., 2005). During the same period, sleep structure also changes, becoming more similar to that of adults: sleep becomes monophasic with gradual reduction in naptime, decreases in rapid eye movement (REM) sleep, and increases in non-REM sleep. Sleep maturation is generally achieved during the preschool years (Bathory and Tomopoulos, 2017; Jiang, 2020).

Given the substantial proportion of time spent sleeping during early childhood (Galland et al., 2012), it is crucial to investigate the effects of sleep quantity and quality on young children’s behavioral adjustment, such as internalizing and externalizing problems, which have persistent impact on later formal schooling (Bornstein et al., 2010). However, preschool-age children’s sleep has received relatively less attention than other age groups’. The majority of prior studies concerning sleep and behavioral adjustment have focused on elementary-level or older students (Becker et al., 2017). Furthermore, there is a dearth of research that examines the impact of maternal depressive symptoms (MDS) on the relationship between children’s sleep and behavioral adjustment among preschoolers. Most of the studies examining associations among MDS, children’s sleep, and children’s adjustment have analyzed cross-sectional data from infants or clinical populations (e.g., Bayer et al., 2007; Armitage et al., 2009; Newland et al., 2016). Therefore, it remains relatively unknown how preschool-age children’s sleep patterns, behavioral adjustment, and MDS are interrelated over time in non-clinical samples.

To address these gaps in the literature, the current study aimed to examine individual trajectories of and longitudinal associations among child sleep (duration and problems), behavioral adjustment [externalizing and internalizing behaviors (EB and IB)], and MDS over 2 years with preschoolers from community populations.

### Children’s Sleep and Behavioral Adjustment

Sleep duration (SD) is one of the most commonly studied sleep-related variables (Galland et al., 2012) along with sleep problems (SPs; e.g., bedtime resistance, delays in falling asleep, night waking, and sleep walking) as a proxy of sleep quantity and quality, respectively (Fisher and McGuire, 1990). SPs are quite prevalent in early childhood and tend to gradually decline from infancy to middle childhood: SP incidence in early childhood ranges from a fourth (e.g., Blader et al., 1997) to a seventh of the samples (e.g., Martin et al., 2007), with lower incidence found among school-aged children (11%–15%, 6–12 years; Owens et al., 2000; Mindell et al., 2009). During early childhood, the types of SPs also change, with night awakening and difficulty falling asleep being more common among 1–5 and 6–11 year olds, respectively (Williamson et al., 2019).

Inadequate quality or quantity of sleep has been reported to exert negative impact on young children’s emotional and behavioral development concurrently and longitudinally (Goodnight et al., 2007; Tso et al., 2016; Williams et al., 2016; Cremone et al., 2018; Williamson et al., 2020). Children who had insufficient sleep were found to respond more readily with anger and sadness to social cues compared to those who did not (Leotta et al., 1997; Saghir et al., 2018). Insufficient sleep quantity has been associated with externalizing and internalizing problems (Yokomaku et al., 2008; LeBourgeois et al., 2013; Tso et al., 2016; Bélanger et al., 2018). For example, children with shortened SD tended to display more hyperactive and inattentive responses as well as less prosocial behaviors (Tso et al., 2016; Bélanger et al., 2018). A similar tendency has been observed between nighttime SPs (e.g., night waking and settling problems) and maladaptive behaviors (Gregory and O’Connor, 2002; Williams et al., 2016; Quach et al., 2018; Reynaud et al., 2018; Williamson et al., 2020). Children who had higher than average incidence of SPs during the first 5 years of life tended to be more hyperactive, exhibited more emotional problems, and were less prosocial and self-regulatory at 6–7 years of age (Williams et al., 2016). Furthermore, children who exhibited persistent SPs until lower elementary grades showed higher levels of externalizing and internalizing problems at 10–11 years of age (Williamson et al., 2020).

In this sleep-adjustment association, sleep is assumed to play a leading role by affecting self-regulation, which is involved in daytime social functioning (Dahl, 1996). Sleep reinvigorates the brain by granting it rest, thereby allowing adequate levels of alertness and arousal during the daytime. In addition, sleep re-synchronizes multiple brain regions, facilitating efficient processing of information and communication among them (Dahl, 1996; Fattinger et al., 2017). Sleep deprivation itself is a stress on the body, resulting in increased amygdala activation and secretion of stress hormones, such as epinephrine and cortisol (Hirotsu et al., 2015), which are known to lead to deactivation of the prefrontal cortex involved in the generation and control of emotional responses (Goldstein and Walker, 2014; Bathory and Tomopoulos, 2017). Thus, through these paths, SPs and decreased SD are assumed to negatively affect children’s self-regulation, thereby causing problems in behavioral adjustment.

Taken together, SD and SPs seem to be closely linked to children’s daytime behavioral adjustment, probably mainly through self-regulation depletion. However, previous research has not investigated how changes in preschool-age children’s sleep and adjustment are interrelated. The influence of MDS on the relationship between children’s sleep and behavioral adjustment has not been addressed either, despite the fact that child sleep and adjustment problems are susceptible to stress-generating (e.g., maternal depression, marital conflict, and poverty) and compensating factors within a family (e.g., responsive parenting and parental education). Thus, this study focuses on the impact of MDS, considering other domestic factors as covariates in the analysis.

### Maternal Depression and Children’s Sleep and Behavioral Adjustment

Considering the enormous impact that mothers can have on their children’s living and growth during the early years of life, mothers’ unstable and negative affect states, especially depressive symptoms, are expected to exert adverse effects on child development, including sleep and behavioral adjustment. However, findings regarding preschool-age children’s sleep and MDS are inconsistent, partly due to a lack of relevant studies. In one study (Schultz et al., 2020), 4–5-year-old children with depressed mothers tended to sleep significantly less. Also, the association between MDS and children’s sleep was stronger among mothers with moderate to severe levels of symptoms than among those with minimal to mild symptoms. In contrast, in another study (De Jong et al., 2016), no associations were found between MDS and children’s total SD, while higher levels of MDS were related to greater variability in child SD. Regarding SPs, previous research on non-clinical samples mostly focused on elementary school children (e.g., Buckhalt et al., 2009; Kelly and El-Sheikh, 2011; El-Sheikh et al., 2012), overall showing positive associations between MDS and child SPs.

Moreover, cross-sectional research has consistently established the link between MDS and child behavioral adjustment (e.g., Dawson et al., 2003; Gartstein and Fagot, 2003; Koblinsky et al., 2006; Goodman et al., 2011). Only a small number of studies have investigated this relationship longitudinally (Cents et al., 2013; Giallo et al., 2015; Park et al., 2018; Pietikäinen et al., 2020). For instance, Pietikäinen et al. (2020) examined both maternal and paternal depressive symptoms during the first 2 years after childbirth and their relationship with children’s internalizing and externalizing problems at 2 and 5 years of age. They found that persistent MDS were related to children’s internalizing and externalizing problems at both measurement points, while paternal depressive symptoms did not independently predict child behavioral adjustment. In another study (Cents et al., 2013), similar patterns were observed from mid-pregnancy to 3 years: children with mothers displaying increased depressive symptoms throughout the years exhibited significantly more internalizing and externalizing problems than those whose mothers’ showed decreased depressive symptoms.

Biological and environmental factors may explain the paths through which MDS affect children’s sleep and behavioral adjustment and vice versa. First, mothers and children shared genes can modulate neurotransmitters involved in sleep and emotional regulation (e.g., norepinephrine and serotonin; Jiang, 2020). Second, irresponsive parenting and insecure attachment resulting from MDS are assumed to contribute to the MDS-child sleep association. MDS are known to be frequently associated with disrupted parenting and fewer attempts of mother–child interactions with overall lower levels of affective, cognitive, and social stimulations (Lovejoy et al., 2000; Teti and Crosby, 2012). These parenting practices are likely to evoke the child’s frustration resulting from persistent dissatisfaction of basic needs inducing stress responses (Ulmer-Yaniv et al., 2018). Chronic stress can lead to disorders in emotional regulation (Marusak et al., 2015), the negative effects of which are likely to be more severe, especially during the early period of life because of the higher brain plasticity (Shonkoff and Phillips, 2000). At the same time, considering that children’s greater behavioral problems can contribute to mothers’ negative mood state eliciting greater levels of caring burden and stress responses (Meltzer and Mindell, 2007; Wagner and Valdez, 2020), MDS and children’s behavioral problems [externalizing and internalizing behaviors (EB and IB)] are apt to reinforce each other, creating a vicious cycle (Warren et al., 2006).

The impact of MDS on children’s sleep and behavioral adjustment during the preschool period is not likely to be less important than during infancy and other developmental stages. Nevertheless, the vast majority of relevant studies has focused on the impact of postpartum depressive symptoms in the first few months to 3 years after childbirth (e.g., Ystrom et al., 2017; Parade et al., 2019; Cook et al., 2020; Asmussen et al., 2021). Given the importance of early childhood as a transition from infancy to middle childhood in both sleep and behavioral adjustment and the observed developmental changes during this period, understanding how these processes change over time in relation to MDS is crucial. However, few studies have considered the associations among longitudinal trajectories in children’s sleep, behavioral adjustment, and MDS, especially focusing on the changes in each variable. Thus, the present study aims to investigate the following research question: *how are the trajectories of these variables and their longitudinal associations*? This study addresses the question with three waves of national-level data using latent growth modeling (LGM). This allows to demonstrate individual trajectories of sleep measures (duration and problems), behavioral adjustment, and MDS as well as longitudinal change dynamics during the preschool period.

## Materials and Methods

### Participants

Data were obtained from the National Panel Study on Korean Children (PSKC). The PSKC has collected annual information from a nationally representative sample of approximately 1,700 children and their families in Korea since 2008. The present study used data from 2012 to 2014 (T_1_, T_2_, and T_3_) that spanned across 3 years of preschool, from ages 4–6. A total of 1,703, 1,662, and 1,620 children and their mothers participated at T_1_, T_2_, and T_3_, respectively. Children’s ages were approximately 48–54 months (boys: 51%; girls: 49%), 60–66 months (boys: 51.5%; girls: 48.5%), and 72–79 months (boys: 51.4%; girls: 48.6%) at T_1,_ T_2_, and T_3_, respectively. Main caregivers (mostly mothers, T_1_: 99.9%, T_2_: 99.8%, and T_3_: 99.8% of the total respondents) answered on a questionnaire concerning main and confounding variables at the three measurement points, respectively, except negative emotionality (only at T_1_) and marital conflict (at T_1_ and T_3_).

Complete data, with no missing information on main and confounding variables, were available for 1,635 (76%), 1,606 (74.7%), and 1,559 (72.51%) cases at T_1_, T_2_, and T_3_, respectively. Throughout the three time points, the parents [98.6% (T_1_), 98% (T_2_), and 97.6% (T_3_)] remained *married* and less than 1% of the families were from ethnic minority groups. Additionally, approximately 70% of the parents had two or more years of college education and the rest had completed high school.

### Measures

#### Internalizing and Externalizing Behaviors

Information on children’s adjustment outcomes was collected using the Child Behavior Checklist for ages 1.5–5 (CBCL/1.5–5), which was validated and standardized in a Korean sample (Oh and Kim, 2009). The same version was also applied at age 6 (T_3_) for continuity of measurement and appropriateness of content, since the sampled children did not enter elementary school until T_4_. Mothers rated 100 items related to their child behavior over the past 2 months on a three-point scale ranging from *not true* (0 point) to *very true* or *often true* (2 points). The total score was calculated for the *internalizing domain* [e.g., *emotionally reactive* (nine items), *anxious/depressed* (eight items), *somatic complaints* (11 items), and *withdrawn* (eight items)] and *externalizing domain* [e.g., *attention problems* (five items) and *aggressive behavior* (19 items)]. Cronbach’s alphas for the scales at each time point ranged from 0.57 to 0.88, 0.55 to 0.87, and 0.42 to 0.89 at T_1_, T_2_, and T_3_, respectively.

#### Sleep Problems

Seven items of the CBCL 1.5–5 were used to assess children’s sleep functioning on a three-point scale ranging from *not true* (0 point) to *very true* or *often true* (2 points): (1) does not want to sleep alone, (2) has trouble getting to sleep, (3) nightmares, (4) resists going to bed at night, (5) sleeps less than most children during day and/or night, (6) talks or cries out in sleep, and (7) wakes up often at night. The alpha reliabilities across the three time points were 0.71 at T_1_, 0.60 at T_2_, and 0.84 at T_3_.

#### Sleep Duration

Mothers provided the usual times their children went to sleep and awoke daily through a parental questionnaire (hours and minutes). Since children tend to take naps during the daytime during preschool, SD was calculated as the sum of nocturnal sleep hours (the difference between bedtime and wake-up time) and daytime nap.

#### Maternal Depression

Maternal depression was assessed using the Kessler Psychological Distress Scale (K6; Kessler et al., 2002), which has been validated in the Korean population (Paik, 2010). The scale consists of six items on one’s emotional distress state [e.g., during the past 4 weeks, how much of the time did you feel: (1) so sad that nothing could cheer you up?; (2) nervous?; (3) restless or fidgety?; (4) hopeless?; (5) that everything was an effort?; and (6) worthless?]. Each question was rated on a five-point Likert scale ranging from 1 (*none of the time*) to 5 (*all of the time*). The alpha reliability remained at 0.92 across all measurement time points. A total score was calculated by adding up each item’s score, with higher scores indicating higher levels of maternal depression.

### Covariates

#### Negative Emotionality

Five items from the *emotionality* scale of the Korean version of Buss and Plomin’s (1984) Emotionality, Activity, and Sociability (EAS)—Temperament Survey for Children-Parental Ratings (Huh et al., 2006) were used to collect information on children’s negative emotionality. Mothers rated children’s emotionality (e.g., negative mood, irritability, and intensity of negative reactions) on a five-point scale ranging from *not typical of my child* (1 point) to *very typical of my child* (5 points). Information on negative emotionality was collected only at T_1_ (alpha = 0.75). Individual item’s ratings were added together, with higher scores indicating higher levels of negative emotionality.

#### Responsive Parenting

Information on responsive parenting behaviors was collected using the *Social Interaction* Scale of the Parental Style Questionnaire (PSQ; Bornstein, 1989). This scale evaluates parental *warmth* and *responsiveness*. The items were rated on a five-point scale ranging from *hardly at all* (1 point) to *all the time* (5 points). Total scores were calculated. Alpha reliability values were 0.86, 0.85, and 0.86 at T_1_, T_2_, and T_3_, respectively.

#### Home Environment

Children’s exposure to developmental stimulations through positive domestic environments was examined using the Early Childhood Home Observation for Measurement of the Environment (EC-HOME; Caldwell and Bradley, 2003), which is validated in a Korean context (Kim and Kwak, 2007). The EC-HOME consists of 55 yes or no questions from eight subscales (i.e., learning materials, language stimulation, physical environment, academic stimulation, modeling, variety, and acceptance). The sum of the eight subscales was used in the analyses.

#### Marital Conflict

Perceived marital conflict was assessed using the scale used in the Prevention and Relationship Enhancement Program (PREP; Markman et al., 2001), which is validated in a Korean sample (Yu and Kim, 2005). The scale was developed to diagnose the level of couples’ distress and predict divorce rates on a five-point scale [*hardly at all* (1 point) to *all the time* (5 points)]. Scores on the eight items were included in the analyses. Since items on marital conflict were measured at T_1_ and T_3_, but not at T_2_, the two available data points were averaged [alpha = 0.92 (T_1_) and 0.99 (T_3_)]. Higher scores indicate higher levels of marital conflict.

### Analysis

Latent growth modeling was conducted to examine the *associations* among the trajectories of children’s sleep (SD and SPs), child behavioral adjustment outcomes (internalizing and externalizing problems), and MDS as well as *overall pattern and variability* in developmental trajectory for each variable, while controlling for relevant domestic factors (e.g., responsive parenting, developmental stimulation, and marital conflict) and children’s temperament (negative emotionality; Troxel et al., 2013). In the current analyses, only cases with complete data at all three time points were used (*n* = 1,427). Statistical testing suggested that only minimal bias was introduced by removing cases with missing data. Significant differences were found only sporadically across a few of the main and confounding variables between the groups of participants included and excluded from the analysis: children’s *internalizing problems* at T_3_ (higher in the participants included; *t* = 1.80, *p* < 0.05), *responsive parenting* at T_1_ and T_2_ (higher in the participants excluded; *t* = −1.78, *p* < 0.01; *t* = −2.67, *p* < 0.01), the *home environment* at T_1_ and T_2_ (higher in the participants included; *t* = 4.38, *p* < 0.00; *t* = 2.03, *p* < 0.05), and *marital conflict* at T_1_ and T_3_ (higher in the participants excluded; *t* = −2.16, *p* < 0.05; *t* = −1.75, *p* < 0.05).

First, descriptive statistics and zero-order correlations among the variables were examined using Stata 12. To ameliorate the effects of skewed distributions (Table 1), SPs and internalizing and externalizing problems were log-transformed. Then, univariate growth models were fitted to examine the overall chronological patterns of changes in MDS, children’s sleep, and children’s adjustment. Next, to examine the associations among the variables controlling for relevant covariates, multivariate growth models were specified. Separate models for each of the sleep parameters (i.e., SD and SPs) were set up to detect possible differences in longitudinal trajectories of SD and SPs and their association with children’s adjustment outcomes and MDS. To reduce problems resulting from multicollinearity between internalizing and externalizing problems (Table 2), separate models were fitted for each of them. Thus, in total, four multivariate models were examined as: *Model 1* included SD, externalizing problems, and MDS; *Model 2* included SD, internalizing problems, and MDS; *Model 3* included SPs, externalizing problems, and MDS; and *Model 4* included internalizing problems and MDS. Each model included one growth curve for each construct [SD (SPs), externalizing (internalizing) problems, and MSD] and estimated associations among growth factors (levels and slopes). In all of these models, child negative emotionality (Cremone et al., 2018), responsive parenting (Putnam et al., 2002), home environment, and marital conflict were controlled for as confounding variables. Family socioeconomic status (SES) indicators (i.e., maternal education and family income) and child gender were not controlled in the final models because they had no or very weak correlations with only some of the main variables (Table 2) and the model fit indices dropped when they were included in the models. LGM was executed with Mplus 7.4 (Muthén and Muthén, 2012) with equal spacing between measurement occasions (T_1_, T_2_, and T_3_ were coded as 0, 1, and 2, respectively).

**Table 1:** Descriptive statistics on children’s sleep and adjustment and maternal depression.

	N	M	SD	Min	Max	Skew
*Sleep Duration (SD)*						
SD at T_1_	1703	9.93	0.80	6.01	13.55	0.02
SD at T_2_	1,662	9.88	0.74	7.00	13.00	−0.03
SD at T_3_	1,620	9.80	0.70	7.00	12.50	−0.18
*Sleep Problem (SP)*						
SP at T_1_	1,694	2.07	1.89	0.00	11.00	1.21
SP at T_2_	1,651	1.74	1.69	0.00	12.00	1.36
SP at T_3_	1,605	1.63	1.58	0.00	11.00	1.41
*Externalizing Behaviors (EB)*						
EB at T_1_	1,694	7.77	5.83	0.00	32.00	0.76
EB at T_2_	1,651	6.33	5.52	0.00	32.00	1.06
EB at T_3_	1,605	5.67	5.32	0.00	36.00	1.25
*Internalizing Behaviors (IB)*						
IB at T_1_	1,694	8.42	6.42	0.00	45.00	1.12
IB at T_2_	1,651	7.34	6.28	0.00	42.00	1.45
IB at T_3_	1,605	6.75	6.03	0.00	37.00	1.37
*Maternal Depression (MD)*						
MD at T_1_	1,672	11.76	4.51	6.00	30.00	0.72
MD at T_2_	1,614	11.62	4.43	6.00	30.00	0.79
MD at T_3_	1,565	11.74	4.61	6.00	30.00	0.70

T_1_ = first measurement wave (2012); T_2_ = second measurement wave (2013); T_3_ = third measurement wave (2014); and unit of sleep duration = hours.

**Table 2:** Correlations among the main and socio-demographic variables.

	1	2	3	4	5	6	7	8	9	10	11	12	13	14	15	16	17	18	19	20	21	22	23	24
1. SD T_1_	−	−	−	−	−	−	−	−	−	−	−	−	−	−	−	−	−	−	−	−	−	−	−	
2. SD T_2_	0.38^*^	−	−	−	−	−	−	−	−	−	−	−	−	−	−	−	−	−	−	−	−	−	−	
3. SD T_3_	0.31^*^	0.54^*^	−	−	−	−	−	−	−	−	−	−	−	−	−	−	−	−	−	−	−	−	−	
4. SP T_1_	−0.09^*^	−0.05^*^	−0.06^*^	−	−	−	−	−	−	−	−	−	−	−	−	−	−	−	−	−	−	−	−	
5. SP T_2_	−0.05	−0.06^*^	−0.04	0.50^*^	−	−	−	−	−	−	−	−	−	−	−	−	−	−	−	−	−	−	−	
6. SP T_3_	−0.03	−0.04	−0.00	0.41^*^	0.52^*^	−	−	−	−	−	−	−	−	−	−	−	−	−	−	−	−	−	−	
7. EB T_1_	−0.09^*^	−0.01	−0.03	0.49^*^	0.28^*^	0.28^*^	−	−	−	−	−	−	−	−	−	−	−	−	−	−	−	−	−	
8. EB T_2_	−0.06^*^	−0.05^*^	−0.03	0.29^*^	0.48^*^	0.32^*^	0.59^*^	−	−	−	−	−	−	−	−	−	−	−	−	−	−	−	−	
9. EB T_3_	−0.08^*^	−0.02	−0.04	0.27^*^	0.33^*^	0.46^*^	0.55^*^	0.65^*^	−	−	−	−	−	−	−	−	−	−	−	−	−	−	−	
10. IB T_1_	−0.05	−0.01	−0.01	0.55^*^	0.36^*^	0.33^*^	0.70^*^	0.43^*^	0.40^*^	−	−	−	−	−	−	−	−	−	−	−	−	−	−	
11. IB T_2_	−0.02	−0.03	−0.02	0.33^*^	0.56^*^	0.37^*^	0.42^*^	0.72^*^	0.48^*^	0.57^*^	−	−	−	−	−	−	−	−	−	−	−	−	−	
12. IB T_3_	−0.02	0.01	0.03	0.30^*^	0.39^*^	0.54^*^	0.41^*^	0.50^*^	0.73^*^	0.54^*^	0.63^*^	−	−	−	−	−	−	−	−	−	−	−	−	
13. MD T_1_	−0.06^*^	−0.07^*^	−0.03	0.19^*^	0.16^*^	0.16^*^	0.27^*^	0.23^*^	0.25^*^	0.27^*^	0.22^*^	0.23^*^	−	−	−	−	−	−	−	−	−	−	−	
14. MD T_2_	−0.05^*^	−0.05	−0.03	0.12^*^	0.18^*^	0.21^*^	0.22^*^	0.25^*^	0.23^*^	0.22^*^	0.26^*^	0.24^*^	0.55^*^	−	−	−	−	−	−	−	−	−	−	
15. MD T_3_	−0.07^*^	−0.04	−0.04	0.17^*^	0.21^*^	−0.10^*^	0.22^*^	0.25^*^	0.27^*^	0.23^*^	0.25^*^	0.29^*^	0.54^*^	0.56^*^	−	−	−	−	−	−	−	−	−	
16. RP T_1_	0.04	0.05^*^	0.03	−0.10^*^	−0.09^*^	−0.09^*^	−0.26^*^	−0.26^*^	−0.25^*^	−0.19^*^	−0.19^*^	−0.19^*^	−0.33^*^	−0.26^*^	−0.28^*^	−	−	−	−	−	−	−	−	
17. RP T_2_	0.06^*^	0.07^*^	0.03	−0.13^*^	−0.09^*^	−0.08^*^	−0.25^*^	−0.28^*^	−0.24^*^	−0.18^*^	−0.20^*^	−0.17^*^	−0.21^*^	−0.24^*^	−0.23^*^	0.64^*^	−	−	−	−	−	−	−	
18. RP T_3_	0.06^*^	0.05	−0.02	−0.10^*^	−0.12^*^	−0.15^*^	−0.20^*^	−0.28^*^	−0.29^*^	−0.18^*^	−0.22^*^	−0.24^*^	−0.26^*^	−0.25^*^	−0.33^*^	0.60^*^	0.59^*^	−	−	−	−	−	−	
19. ME	0.04	−0.04	−0.02	0.06^*^	0.05^*^	0.03	−0.08^*^	−0.03	−0.09^*^	−0.01	0.01	−0.02	−0.08^*^	−0.05^*^	−0.07^*^	0.13^*^	0.16^*^	0.13^*^	−	−	−	−	−	
20. FE	−0.05^*^	−0.06	−0.05	−0.01	−0.01	−0.02	0.01	0.00	−0.05	−0.01	−0.02	−0.05	−0.01	−0.03	0.02	−0.02	0.00	0.01	0.02	−	−	−	−	
21. NE	−0.06^*^	−0.02^*^	−0.05	0.24^*^	0.21^*^	0.22^*^	0.44^*^	0.36^*^	0.36^*^	0.43^*^	0.33^*^	0.36^*^	0.30^*^	0.24^*^	0.21^*^	−0.27^*^	−0.21^*^	−0.25^*^	−0.03	0.00	−	−	−	
22. Income T_1_	−0.01	0.01	0.03	−0.07^*^	−0.04	−0.02	−0.06^*^	−0.02	−0.03	−0.08^*^	−0.06^*^	−0.06^*^	−0.04	−0.04	−07^*^	0.03	0.08^*^	0.04	0.16^*^	−0.00	−0.03	−	−	
23. Income T_2_	0.01	−0.07^*^	−0.06^*^	−0.06^*^	−0.04	−0.04	−0.09^*^	−0.06^*^	−0.07^*^	−0.10^*^	−0.08^*^	−0.08^*^	−0.08^*^	−0.10^*^	−0.07^*^	0.07^*^	0.12^*^	0.06^*^	0.30^*^	−0.00	−0.06^*^	0.60^*^	−	
24. Income T_3_	−0.02	−0.07^*^	−0.07^*^	−0.05^*^	−0.02	−0.07^*^	−0.10^*^	−0.08^*^	−0.08^*^	−0.10^*^	−0.09^*^	−0.10^*^	−0.09^*^	−0.09^*^	−0.11^*^	0.10^*^	0.14^*^	0.08^*^	0.31^*^	0.00	−0.04	0.54^*^	0.81^*^	
25. Child sex	0.07^*^	0.09^*^	0.08^*^	0.00	−0.01	−0.00	−0.12^***^	−0.12^***^	−0.11^***^	−0.02	−0.01	0.01	0.02	−0.00	0.00	0.07^**^	0.05^*^	0.03	−0.02	−0.00	0.02	0.02	0.02	0.02

SD, sleep duration; SP, sleep problem; EB, externalizing behaviors; IB, internalizing behaviors; MD, maternal depression; RP, responsive parenting; ME, mother’s education; FE, father’s education; NE, negative emotionality; and child sex, 1 = boys; 2 = girls.

* *p* < 0.05;

** *p* < 0.01; and

*** *p* < 0.001.

## Results

### Descriptive Statistics and Zero-order Correlations

The means and SDs of the variables used in the analyses are presented in Table 1. In average, children’s SD and SPs as well as internalizing and externalizing problems tended to decrease gradually over time. SPs and adjustment outcomes (i.e., internalizing and externalizing problems) were significantly positively correlated within and across waves. However, the associations between SD and adjustment outcomes were relatively weak in magnitude and not all significant. Similar patterns were observed between MDS and sleep variables (SD and SPs) and between MDS and children’s internalizing and externalizing behaviors.

### Univariate Growth Models

The results of the univariate growth model are presented in Table 3. Analyses revealed a significant decrease in children’s SD over time. Children who slept relatively longer at the initial measurement point had a greater decrease in the total amount of daily sleep. The same pattern was found for SPs and adjustment outcomes. Children’s SPs significantly declined over the years, more so among children who initially (T_1_) exhibited more SPs. Similarly, children’s externalizing (internalizing) problems decreased across the 3 years, with greater decreases among those who initially displayed more externalizing (internalizing) problems. MDS did not significantly change over time, and the initial levels of depression were not significantly associated with the degree of change.

**Table 3:** Results of univariate growth curve models.

	Intercept	Slope		Intercept-slope covariance	
	Mean level at T1	Variance	Change per 1-year interval	Variance	
Sleep duration	**9.93(0.02)**	**0.28(0.03)**	**−0.07(0.01)**	**0.09(0.01)**	**−0.06(0.01)**
Sleep problem	**2.01(0.05)**	**1.98(0.15)**	**−0.21(0.02)**	**0.27(0.06)**	**−0.37(0.08)**
Externalizing behaviors	**7.69(0.15)**	**20.90(1.41)**	**−1.05(0.07)**	**1.87(0.59)**	**−1.87(0.69)**
Internalizing behaviors	**8.36(0.16)**	**24.99(1.78)**	**−0.82(0.08)**	**2.32(0.76)**	**−1.84(0.88)**
Maternal depression	**11.78(0.08)**	**10.83(0.91)**	−0.01(0.06)	0.43(0.72)	0.43(0.50)

Values are unstandardized parameter estimates with standard errors in parentheses. Estimates that are significant at *p* ≤ 0.05 or *p* ≤ 0.01 are denoted in bold font.

### Associations of Trajectories of Sleep, Adjustment, and Depression

**Figure 1:**
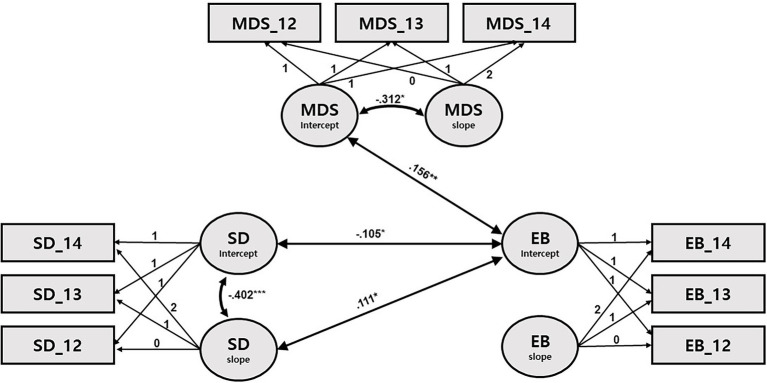
Path diagram for latent growth modeling of sleep duration, externalizing behaviors, and maternal depressive symptoms. SD: sleep duration; EB: externalizing behaviors; and MDS: maternal depressive symptoms. Only significant path coefficients are shown. ^*^p < 0.05, ^**^p < 0.001, and ^***^p < 0.001.

**Figure 2:**
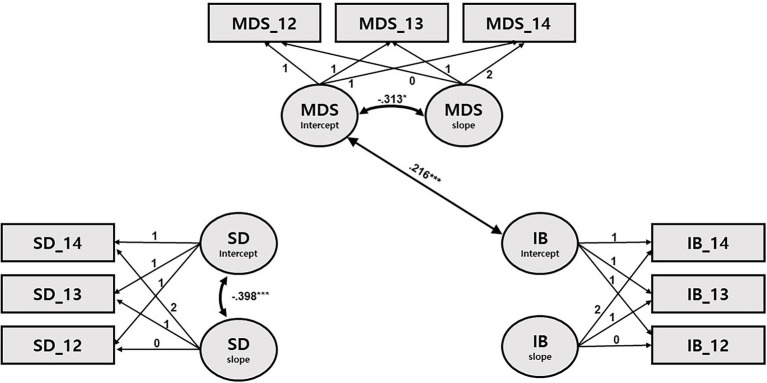
Path diagram for latent growth modeling of sleep duration, internalizing behaviors, and maternal depressive symptoms. SD: sleep duration; IB: internalizing behaviors; and MDS: maternal depressive symptoms. Only significant path coefficients are shown. ^*^p < 0.05, ^**^p < 0.001, and ^***^p < 0.001.

**Figure 3:**
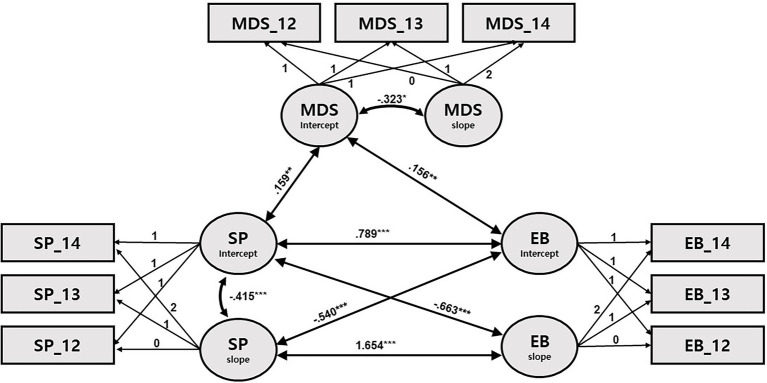
Path diagram for latent growth modeling of sleep problems, externalizing behaviors, and maternal depressive symptoms. SP: sleep problems; EB: externalizing behaviors; and MDS: maternal depressive symptoms. Only significant path coefficients are shown. ^*^p < 0.05, ^**^p < 0.001, and ^***^p < 0.001.

**Figure 4:**
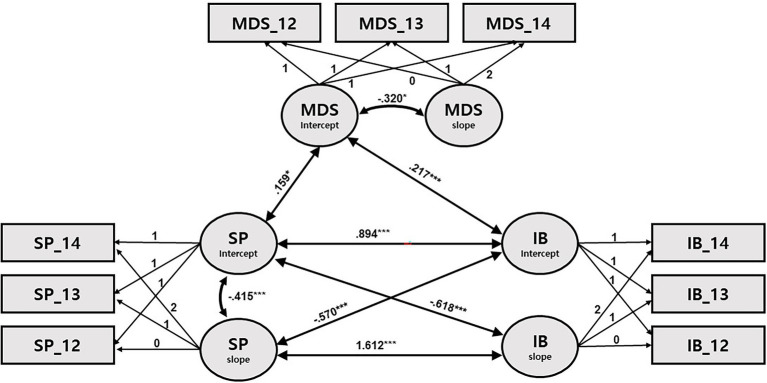
Path diagram for latent growth modeling of sleep problems, internalizing behaviors, and maternal depressive symptoms. SP: sleep problems; IB: internalizing behaviors; and MDS: maternal depressive symptoms. Only significant path coefficients are shown. ^*^p < 0.05, ^**^p < 0.001, and ^***^p < 0.001.

Tables 4–7 and Figures 1–4 present the results of the multivariate growth models. Results of Model 1 and 2 (Tables 4, 5; Figures 1, 2) revealed that initial levels of SD were negatively associated with the initial levels of externalizing behaviors (*β* = −0.105, *p* < 0.05), indicating that the longer (vs. shorter) the initial SD, the lower (vs. higher) the initial externalizing behaviors. However, initial SD was not significantly associated with initial internalizing problems. Next, the initial externalizing problems were positively associated with the slope for SD (*β* = 0.111, *p* < 0.05), indicating that higher (vs. lower) initial levels of externalizing problems were related to slower decline in SD over time. Links between the initial levels of internalizing problems and the slope for SD were only marginally significant (*β* = 0.09, *p* < 0.10). The initial levels of MDS were positively associated with the initial levels of internalizing and externalizing problems (*β* = 0.156, *p* < 0.01; *β* = 0.216, *p* < 0.01), but not with their slope. Children whose mothers had higher levels of depressive symptoms tended to exhibit higher levels of internalizing and externalizing problems at 4 years of age.

**Table 4:** Coefficients among growth factors in multivariate growth model 1: sleep duration—externalizing behaviors.

	1(i1)	2(s1)	3(i2)	4(s2)	5(i3)
	*β*	*β*	*β*	*β*	*β*
*Sleep duration*					
1. Intercept	1				
2. Slope	−0.402^***^	1			
*Externalizing behaviors*					
3. Intercept	−0.105^*^	0.111^*^	1		
4. Slope	0.017	−0.037	−0.099	1	
*Maternal depressive symptoms*					
5. Intercept	−0.087	0.063	0.156^**^	−0.054	1
6. Slope	0.069	−0.016	−0.148	−0.046	−0.312^*^

Model fit indices: RMSEA = 0.036; CFI = 0.977; SRMR = 0.025; and Chi-square = 5031.329 (*p* < 0.00).

* *p* ≤ 0.05;

** *p* ≤ 0.01;

*** *p* ≤ 0.001.

**Table 5:** Coefficients among growth factors in multivariate growth model 2: sleep duration—internalizing behaviors.

	1(i1)	2(s1)	3(i2)	4(s2)	5(i3)
	*β*	*β*	*β*	*β*	*β*
*Sleep duration*					
1. Intercept	1				
2. Slope	−0.398^***^	1			
*Internalizing behaviors*					
3. Intercept	−0.063	0.090^┼^	1		
4. Slope	0.074	−0.053	−0.112	1	
*Maternal depressive symptoms*					
5. Intercept	−0.087	0.064	0.216^***^	−0.084	1
6. Slope	0.068	−0.017	−0.105	0.244	−0.313^*^

Model fit indices: RMSEA = 0.035; CFI = 0.979; SRMR = 0.024; and Chi-square = 4864.974(*p* < 0.00).

┼ *p* ≤ 0.1.

* *p* ≤ 0.05;

*** *p* ≤ 0.001.

**Table 6:** Standardized coefficients among growth factors in multivariate growth model 3: sleep problem—externalizing behaviors.

	1(i1)	2(s1)	3(i2)	4(s2)	5(i3)
	*β*	*β*	*β*	*β*	*β*
*Sleep problem*					
1. Intercept (i1)	1				
2. Slope (s1)	−0.415^***^	1			
*Externalizing behaviors*					
3. Intercept (i2)	0.789^***^	−0.540^***^	1		
4. Slope (s2)	−0.663^***^	1.654^***^	−0.080	1	
*Maternal depressive symptoms*					
5. Intercept (i3)	0.159^**^	−0.043	0.156^**^	−0.056	1
6. Slope (s3)	−0.076	0.230	−0.144	−0.047	−0.323^*^

Model fit indices: RMSEA = 0.058; CFI = 0.952; SRMR = 0.028; and Chi-square = 6003.135(*p* < 0.00).

* *p* ≤ 0.05;

** *p* ≤ 0.01;

*** *p* ≤ 0.001.

**Table 7:** Coefficients among growth factors in multivariate growth model 4: sleep problem—internalizing behaviors.

	1(i1)	2(s1)	3(i2)	4(s2)	5(i3)
	*β*	*β*	*β*	*β*	*β*
*Sleep problem*					
1. Intercept	1				
2. Slope	−0.415^***^	1			
*Internalizing behaviors*					
3. Intercept	0.894^***^	−0.570^***^	1		
4. Slope	−0.618^***^	1.612^***^	−0.082	1	
*Maternal depressive symptoms*					
5. Intercept	0.159^*^	−0.042	0.217^***^	−0.086	1
6. Slope	−0.075	0.230	−0.105	0.251	−0.320^*^

Model fit indices: RMSEA = 0.065; CFI = 0.942; SRMR = 0.027; Chi-square = 6068.377 (*p* < 0.00).

* *p* ≤ 0.05;

*** *p* ≤ 0.001.

Turning to models of SPs (Models 3 and 4; Tables 6, 7; Figures 3, 4), some newly significant associations were detected among growth factors. First, the initial SPs were associated with both the initial levels and the slope of externalizing (*β* = 0.789, *p* < 0.001; *β* = −0.663, *p* < 0.001) and internalizing (*β* = 0.894, *p* < 0.001; *β* = −0.618, *p* < 0.001) problems. These results indicate that the higher the initial levels of SPs, the higher the initial levels of internalizing and externalizing problems; also, the higher the initial levels of SPs, the steeper the decline in internalizing and externalizing problems. In addition, the initial levels of internalizing and externalizing problems were negatively associated with the rate of change in SPs (*β* = −0.540, *p* < 0.001; *β* = −0.570, *p* < 0.001), while the respective slopes of internalizing and externalizing problems were positively associated with the slope of SPs (*β* = 1.654, *p* < 0.001; *β* = 1.612, *p* < 0.001). That is, higher initial levels of internalizing and externalizing problems were associated with greater decreases in SPs, and greater (vs. smaller) decreases in SPs were associated with increased (vs. decreased) decline in internalizing and externalizing problems.

Finally, when mothers’ initial levels of depression were high, its over-time decline was greater in all four models; their children initially had higher levels of SPs and internalizing and externalizing problems only in Models 3 and 4 (Figures 3, 4).

### Model Fit

Model fit was assessed using the root mean square error of approximation (RMSEA), comparative fit index (CFI), and normal theory weighted least squares chi-square (*χ*^2^). For RMSEA, a value less than 0.05 is considered a good fit, and below 0.08 an adequate fit (Kline, 2004); for SRMR, a value below 0.08 is considered a good fit (Hu and Bentler, 1999). By convention, the CFI should be equal to or greater than 0.90 to accept the model. Chi-square was influenced by sample size and indicated a good fit when *p* > 0.05.

## Discussion

This study attempted to examine the developmental trajectories of children’s sleep patterns, behavioral adjustment, and MDS during 3 years among preschoolers (4–6 years of age) and the *associations* among these variables’ growth factors (initial level and rate of change), while controlling for the effects of confounding variables (i.e., responsive parenting, developmental home stimulation, marital conflict, and children’s negative emotionality). The results showed that as children grew, they tended to sleep gradually less and displayed fewer SPs. The over-time reduction in SD and SPs was greater among children who initially, at 4 years old, slept longer and had more SPs. Overall, associations among children’s sleep, behavioral adjustment, and MDS were stronger in the SP than in the SD models. Children’s longer SD at four was significantly associated with lower levels of concurrent externalizing problems, but not with internalizing problems, when controlling for confounding variables. When the levels of externalizing problems at four were higher, a slower decrease in SD was observed until 6 years. Regarding SPs, children with more SPs at four exhibited higher levels of adjustment problems concurrently and a faster decrease in behavioral problems for the next 2 years. The opposite was also the case: when children displayed more adjustment problems at four, the over-time decline in SPs by the age of 6 was faster. In addition, the amount of decrease in SPs was associated with that in children’s internalizing and externalizing problems over time: the greater the decrease in SPs, the greater the reduction in children’s internalizing and externalizing problems. Finally, when mothers displayed higher levels of depressive symptoms, children exhibited higher levels of SPs and internalizing and externalizing behaviors concurrently at four. However, no significant associations were detected between the rates of change in MDS and those in children’s sleep parameters (duration and problems) and internalizing and externalizing problems.

The present study findings on the trajectories of sleep parameters correspond with the prior finding that sleep duration and problems tend to decrease gradually across early childhood, including the preschool period (Petit et al., 2007; Galland et al., 2012; Vriend et al., 2012; Kocevska et al., 2017; Williamson et al., 2019). According to a meta-analysis of sleep research (Galland et al., 2012); daily SD decreases approximately 7.8 min per year between 1 and 4 years of age and 5.9 min per year from 5 to 12 years of age. In the current study, daily SD decreased about 3 min per year between T_1_ and T_2_ and about 4.8 min per year between T_2_ and T_3_. These patterns differ from Galland et al. (2012) in that the decrease was smaller initially and greater in the last years, although the general tendency was similar. This discrepancy might be due to the shorter average sleep hours reported among Asian children compared to their Caucasian counterparts (Liu et al., 2005; Mindell et al., 2010) or to the reliance on parental reports regarding children’s sleep hours.

The finding that shorter SD and more SPs at four were concurrently related with more behavioral adjustment problems is in line with well-established associations in the extant literature (Yokomaku et al., 2008; LeBourgeois et al., 2013; Tso et al., 2016; Williams et al., 2016; Bélanger et al., 2018). The current study contributes further to the literature by revealing the over-time synchrony in the trajectories of SPs and behavioral adjustment outcomes. The greater the reduction in SPs, the larger the decline in behavioral problems and vice versa. The amount of SPs at four was associated with the amount of decline in behavioral adjustment over the next 2 years and vice versa. In these reciprocally concurrent and prospective associations, the impact of SPs on the change in adjustment behaviors was found to be slightly greater than the impact of adjustment behaviors on SPs, implying that sleep plays a primary role in its relationship with behavioral adjustment, probably through emotional regulation, as reviewed earlier (Dahl, 1996). In another longitudinal study examining SPs and emotional and attentional self-regulation from infancy to 8–9 years of age, the magnitude of the paths from SPs to emotional regulation was stronger than the other way around (Williams et al., 2015).

The observed synchronized change in SPs and internalizing and externalizing behaviors is likely explained by neurobiological mechanisms that generate less efficient functions and/or delayed maturation of the brain regions involved in sleep and emotional regulation (e.g., prefrontal cortex and the limbic system, including the amygdala, hypothalamus, and thalamus; Goldstein and Walker, 2014; Bathory and Tomopoulos, 2017). Self-regulation, in particular emotional regulation, has been thought of as a potential mediating factor in the sleep-adjustment association (Dahl, 1996) evidenced by links between inadequate sleep and higher negative (lower positive) emotions as well as the common co-occurrence of affective disorders and sleep abnormalities (Goldstein and Walker, 2014; Bathory and Tomopoulos, 2017). Recent neuroimaging studies with adults found that amygdala activation was 60% greater after sleep deprivation (Yoo et al., 2007). In addition, connectivity between the amygdala and prefrontal cortex was found to decrease after sleep deprivation, which implies impaired self-regulation (Yoo et al., 2007). Also, children with SPs seem to experience additional disadvantages in terms of prefrontal cortex maturation, being manifested as thinner cortex in the dorsolateral prefrontal area (Buchmann et al., 2011; Kocevska et al., 2017). More research is needed to identify the neurological mechanisms underlying disorders of sleep and emotional regulation, but interactions between the environment (e.g., parenting) and genes seem to be one of the causes. Previous research (Davis et al., 2017) demonstrated the moderating effects of parenting on the relationship between child sleep outcomes and genes (e.g., 5-HTTLPR and DRD4) pertaining to both sleep quality and emotional regulation (Bouvette-Turcot et al., 2015; Dutta, 2020): children carrying the short allele(s) of the 5-HTTLPR gene displayed significantly fewer sleep problems at low levels of maternal parenting stress while exhibiting more sleep problems at high levels of maternal parenting stress, supporting the differential susceptibility hypothesis (Ellis et al., 2011). It is possible that the observed interconnections between sleep measures and behavioral adjustment outcomes in the current study (e.g., synchronized greater decrease in SPs and externalizing/internalizing behaviors) might have resulted from the workings of these genes and MDS moderation of the sleep-adjustment relations, although this was not examined in this study.

Given that previous studies have reported that shorter SD and more SPs are associated with greater adjustment problems (also found in this study), it was expected that a faster decrease in SD and slower decline in SPs would be related to more behavioral problems. However, the opposite was found in the present study: higher levels of behavioral adjustment problems at four were associated with a slower decrease in SD and faster decline in SPs in the next 2 years. These seemingly puzzling results seem to arise from the fact that *longer* SD and *greater* SPs at four were related to *faster* reduction in each of the variables over the next 2 years. Since there were concurrent *negative* and *positive* associations between behavioral adjustment and SD and SP, respectively, and both SD and SP showed *greater* decline when their initial levels were higher, behavioral adjustment at four was associated with a *slower* decrease in SD and *faster* decline in SP. Thus, future research should investigate how common it is that preschoolers with initially longer SD and more SPs exhibit relatively faster reduction in both SD and SPs. In addition, whether relationships between initial levels and the amount of change in SD and SP differ depending on children’s disparate levels of behavioral adjustment problems is worth studying. Unfortunately, to the best of my knowledge, no studies have reported how initial levels of SD and SPs are related to their own decrease rate over time in relation to children’s levels of behavioral or emotional regulation. Instead, one study (Williams et al., 2016) examined correlations between SPs at adjacent measurement points throughout the early childhood period and reported a gradual increase in the magnitudes of the correlations over a period of 6–7 years. This differs from the current study’s findings; thus, more research is needed.

A comparison between the SD and SPs models suggests that what matters in predicting children’s behavioral adjustment in relation to MDS is likely *the quality*, rather than *the total amount* of sleep (Bélanger et al., 2018). First, a greater number of associations were detected between SPs than between SD and other variables. While both the initial level (intercept) and the degree of change (slope) were associated with those of behavioral adjustment in the SPs models, only the initial levels of externalizing problems were related to the growth factors of the SD, with no associations found in the internalizing problems model. In addition, the associations between behavioral adjustment (i.e., internalizing and externalizing problems) and SPs were stronger compared to those with SD. These results support the likelihood that sleep quality, rather than quantity propels behavioral adjustment, in accordance with the reviewed literature. Indeed, maladaptive outcomes caused by sleep disruption in adults were found not to be ameliorated by sleep quantity (e.g., Haus and Smolensky, 2006; Genzel et al., 2013).

The associations between MDS, sleep, and behavioral adjustment were not as robust as those between sleep and behavioral adjustment but were more apparent in the SPs models than in SD models. No direct association was found between SD and MDS in this study. Previous research has yielded inconsistent findings, with some showing no associations, as in the current research (e.g., De Jong et al., 2016) and others revealing significant links (e.g., Schultz et al., 2020) between SD and MDS. Concerning SPs, MDS at four were positively associated with the concurrent levels of SPs, in line with the prior findings among elementary school children (e.g., Buckhalt et al., 2009; Kelly and El-Sheikh, 2011; El-Sheikh et al., 2012). Similar to child sleep parameters, MDS was weakly concurrently associated with behavioral adjustment (internalizing and externalizing problems) at four, similar to findings from meta-analytic studies examining the effects of MDS on children’s psychosocial functioning, including internalizing and externalizing problems (Connell and Goodman, 2002; Goodman et al., 2011). In these studies, the effect sizes were small in magnitude, comparable to the current study results (internalizing: *r* = 0.16–0.23; externalizing: *r* = 0.14–0.21). In the present study, the associations between MDS and internalizing problems were slightly stronger than those with externalizing problems, which seems sensible given that *internalizing* problems directly concern emotional reactivity, anxiety, depression, social withdrawal, etc. Regarding children’s externalizing problems, the observed associations might have arisen from reciprocity in a mother–child relationship (Warren et al., 2006): (1) from a mother to a child through fewer demonstrations of emotionally regulated behaviors, less provision of instructions and strategies for behavioral and emotional regulation, and fewer expressions of warmth and affection toward a child (Lovejoy et al., 2000; Eisenberg et al., 2003; Coyne and Thompson, 2011; Wagner and Valdez, 2020); (2) from a child to a mother through relatively higher frequencies of problematic and act-out behaviors, which increases a depressed mother’s psychological burden and fatigue (Meltzer and Mindell, 2007; Wagner and Valdez, 2020).

However, no significant prospective link was found between the levels of MDS at four and the rate of change in child behavioral problems, which partially contradicts previous studies reporting that MDS profiles predicted later child adjustment outcomes (Cents et al., 2013; Giallo et al., 2015; Park et al., 2018). This absence of predictive interplay might be because a comprehensive set of confounding variables, including responsive parenting and negative emotionality, was controlled for in the analysis. In addition, because the study data were collected from the general population and not a clinical sample, the number of mothers and children in the sample with severe depressive symptoms and behavioral problems is likely small to detect an effect. Although no direct link was found between MDS at four and the rate of change in child behavioral problems, an indirect path can be assumed given MDS’s concurrent associations with SPs and behavioral adjustment, each of which significantly predicted the other’s degree of over-time change. Thus, the results of the present study suggest that variations in maternal mental health, even within the non-clinical range, are linked to children’s SPs and adjustment in both direct and indirect ways, even when the effects of diverse family contextual covariates are controlled for.

Taken together, the current study findings imply that during the preschool years, *SD* has some impact on children’s behavioral adjustment, but as children grow, *SPs* seem to be more important in predicting and, thus, probably preventing adjustment problems. While the relationship between SPs and behavioral adjustment seemed to be reciprocal, the magnitude of the path from SPs at four to the rate of change in behavioral adjustment in the next 2 years was somewhat greater than that of the other way around. This finding reinforces the importance of the *quality of sleep* for better socio-emotional developmental outcomes. Therefore, improving sleep quality by addressing any problems in the child’s environment as well as practices within the family, especially focusing on the overall parenting quality, may be an effective approach to improve young children’s general development and health. MDS were found to have a concurrent impact on children’s sleep problems and behavioral adjustment outcomes during the preschool period when children’s dependence on mothers declined as compared to infancy. Mothers (or any main caregivers) are, in general, the most influential entity in child development during the first few years. The current study findings, along with previous work showing that high-quality maternal parenting impact some genes’ phenotypes intervening in sleep and self-regulation, also tells us that keeping mothers’ mental health in check during the immediate postpartum period and throughout the later years, should be the focus of parent support policies and parent education.

## Limitations and Future Research

Despite the merits of this study, some limitations should be noted. First, recent studies have shown that the daily amount of media exposure (screen time) relates to sleep quantity and quality (Garrison et al., 2011; Tso et al., 2016; Zhang et al., 2020) and psychosocial and emotional problems among young children (Pagani et al., 2010; Hinkley et al., 2014). However, this study could not control for the effects of media exposure due to a lack of information. Thus, future studies should consider children’s daily media consumption. Second, this study relied on parental reports on the main study variables, including sleep quantity and quality, suggesting the possibility of slight over- or under-estimation of the real sleep time and problems, although parental reports have shown generally high agreement with objective measures such as actigraphy (Sadeh, 1994; Tikotzky and Sadeh, 2001; Sekine et al., 2002). The possibility that depressed mothers might have perceived their children’s sleep and behavioral problems more severely also necessitates more studies using objective measures concerning preschool period. Next, even though this study tried to comprehensively control for diverse domestic factors, other aspects of family functioning were not represented in this study, in particular, parental practices pertaining to sleep, such as co-sleeping, bedtime routines, sleep hygiene (Arora, 2019), and other related factors like nutrition and daily exercises (Westerlund et al., 2009; Łuszczki et al., 2021). Similarly, the effects of neurobiological factors, such as mother–child shared genetic makeup and mechanisms intervening in sleep and behavioral adjustment independently and jointly, were not considered in the current study. Thus, more research addressing relevant factors at various levels—from biological to environmental (e.g., genes, maturation, relevant brain regions functioning, and daily activities including childcare-related variables)—, would contribute to connecting the dots and completing the whole picture.

## Data Availability Statement

The original contributions presented in the study are included in the article/supplementary material; further inquiries can be directed to the corresponding author.

## Ethics Statement

This study used a national-level open data set. Written informed consent to participate in this study was provided by the participants’ legal guardian/next of kin.

## Author Contributions

The author confirms being the sole contributor of this work and has approved it for publication.

## Conflict of Interest

The author declares that the research was conducted in the absence of any commercial or financial relationships that could be construed as a potential conflict of interest.

## Publisher’s Note

All claims expressed in this article are solely those of the authors and do not necessarily represent those of their affiliated organizations, or those of the publisher, the editors and the reviewers. Any product that may be evaluated in this article, or claim that may be made by its manufacturer, is not guaranteed or endorsed by the publisher.
